# Stress awareness and decision-making under uncertainty: Gender-specific effects of mild hypoxia in the Iowa Gambling Task

**DOI:** 10.3758/s13415-025-01320-1

**Published:** 2025-06-23

**Authors:** S. Pighin, A. Fornasiero, M. Testoni, A. Bogani, N. Bonini, B. Pellegrini, F. Schena, L. Savadori

**Affiliations:** 1 Center for Mind/Brain Science, University of Trento, Corso Bettini, 31 38068 Rovereto, TN Italy; 2 CeRiSM, Sport Mountain and Health Research Centre, University of Verona, Rovereto, Italy; 3 Department of Engineering for Innovation Medicine, University of Verona, Verona, Italy; 4 Department of Neuroscience, Biomedicine and Movement, University of Verona, Verona, Italy; 5 Department of Economics and Management, University of Trento, Trento, Italy

**Keywords:** Decision making, Mild hypoxia, Stress, Gender differences, Risk-taking behavior, Iowa Gambling Task

## Abstract

Decision-making under uncertainty is a key cognitive function that is sensitive to acute stress. While prior studies have documented gender-specific effects of stress (i.e., typically increased risk-taking in males and greater caution in females), such findings have primarily emerged in conditions where participants were aware of the ongoing stressor. The present study explored whether stress awareness modulates gender differences in risk-taking by manipulating participants’ awareness of being under mild hypoxia (i.e., reduced oxygen availability), a systemic stressor that often goes unnoticed by individuals. Sixty-four participants completed the Iowa Gambling Task (IGT) under normoxic (fraction of inspired oxygen (FiO_2_) = 20.9%) and mildly hypoxic (FiO_2_ = 14.1%) conditions, with participants either being informed or uninformed about the stressor. Results indicated that when participants were aware of the stressor, males exhibited an increased selection of disadvantageous decks (corresponding to greater risk-taking in the IGT) under mild hypoxia, whereas females demonstrated a trend toward more cautious and advantageous choices. In contrast, when unaware of the stressor, both genders showed similar, modest increases in disadvantageous choices under hypoxia. These findings suggest that gender-specific coping strategies in risky decision-making are triggered by the conscious appraisal of stress, rather than by its mere physiological presence, and support theoretical models emphasizing the role of cognitive appraisal and internalized gender norms in shaping decision-making under stress.

## Introduction

Making decisions under acute stress has become a pervasive aspect of daily life, often occurring regardless of individuals’ awareness. Stress, indeed, can take distinct forms. It can be consciously experienced, as when we realize that we are under pressure due to an imminent deadline, an interpersonal conflict, or an emergency situation. At other times, stress may remain outside of our conscious awareness, still acute and physiologically impactful, yet unnoticed, such as when subtle environmental stressors influence us without our realization, silently shaping our behaviors, emotions, and decisions.

In this article, we analyze the role of stress awareness in decision-making under uncertainty and examine how this effect interacts with gender, exploring whether males and females respond differently to an environmental stressor depending on their awareness of being under stress. Manipulating stress awareness presents significant methodological challenges and, to the best of our knowledge, this is the first study specifically designed to address this issue. To achieve this goal, we have employed an environmental stressor, mild hypoxia, which is a controlled mild reduction of the oxygen concentration in the air. The manipulation of this specific stressor offers the advantage of significantly altering individuals’ physiological and cognitive responses without the stress being consciously perceived (Pighin et al., 2020, 2022), thereby enabling the examination of the effects of stress awareness within a controlled setting.

Acute stress – a short-term physiological and psychological response to a perceived threat or challenge – generally causes individuals to make economically disadvantageous decisions, supposedly due to an increase in risk-taking and a greater search for rewards, in contexts where such behaviors are counterproductive (Porcelli & Delgado, 2017; Starcke & Brand, 2016). Acute stress has been shown to increase tolerance to uncertainty even in contexts where highly uncertain options are associated with high payoffs, highlighting a potentially beneficial effect under specific conditions (Byrne et al., 2021). Acute stress also appears to alter individuals’ emotional responses, reducing positive emotions in favor of negative ones, and it was found to significantly affect neural activity, particularly in areas related to reward processing and cognitive control (Liu et al., 2024; Porcelli & Delgado, 2017).

Of particular relevance to the present study, acute stress seems to influence individuals differently depending on their gender. For example, females tend to become more cooperative under stress, accepting unfair offers, while males do not significantly change their behavior (Youssef et al., 2018). In general, acute stress amplifies gender differences in decision-making under conditions of uncertainty: males tend to take more risks, while females become more risk-averse (e.g., Mather & Lighthall, 2012). This gender-specific response has been observed both in decision-making contexts where risk-taking is associated with economically disadvantageous outcomes (e.g., in the Iowa Gambling Task; Preston et al., 2007; van den Bos et al., 2009) and in decision-making contexts where risk-taking is associated with greater gains (e.g., in the Balloon Analogue Risk Task; Lighthall et al., 2009, 2012; Pighin et al., 2015).

Gender differences in risk-taking under acute stress have been attributed to a combination of evolutionary, neurobiological, and sociocultural factors. Evolutionary theories propose that, in ambiguous situations, risk aversion may have benefited females by enhancing resource stability and offspring survival, while males may have evolved a greater propensity for risk-taking in competitive or exploratory contexts (Taylor et al., 2000; Wilson & Daly, 1985). This difference in risk attitude is supported by neurobiological evidence, with gender-specific activation patterns observed in the dorsal striatum – an area crucial for reward processing and habit formation (e.g., Lighthall et al., 2012; Porcelli et al., 2012), as well as by hormonal influences, where testosterone is associated with increased risk-taking and estrogen with greater uncertainty aversion (for a review, see Apicella et al., 2015; Kurath & Mata, 2018). Finally, sociocultural factors, including gender role stereotypes, also seem to further shape these differences, as individuals may conform to socially reinforced norms about risk-taking behavior under stress (for a review, see Ellemers, 2018).

A common limitation in studies that have found gender differences in decision-making under uncertainty is that participants were always aware of the stressful condition to which they were exposed. This approach does not allow researchers to determine whether gender-specific differences in acute stress responses are driven by the cognitive appraisal of the ongoing stressor or stem from more generalized physiological mechanisms. Indeed, previous studies have employed either processive stressors (i.e., factors that activate a stress response through higher-order cognitive and emotional regulation mechanisms, such as time or social pressure), or systemic stressors (i.e., factors that trigger an immediate physiological response through low-order neural circuits) exerting a state of consciously perceived discomfort (e.g., the Cold Pressure Test, in which participants immerse their hand in ice-cold water). Notably, studies reporting gender differences have not used non-conscious stressors, and the only study employing an undetected stressor (i.e., mild hypoxia) found no gender differences in decision-making under uncertainty (Pighin et al., 2020). In that study, risk-taking under uncertainty was examined under mild hypoxia, a stressor that, due to its unique characteristics, went unnoticed by participants. Participants completed the Balloon Analogue Risk-Taking Task (BART) under both normoxic (fraction of inspired oxygen (FiO_2_) equal to 20.9%) and mildly hypoxic (FiO_2_ equal to 14.1%) conditions. The results indicated that participants took more risks in the mildly hypoxic environment (that is the stressful condition) than in the normoxic one, with no observed gender differences. As acknowledged by the authors, the absence of a significant interaction between gender and stress may be due to methodological limitations, primarily a small sample size. However, this finding may also indicate that gender differences in risk-taking behavior depend on the specific nature of the stress manipulation. In particular, the cognitive appraisal of the stressor could play a critical role in shaping gender-specific responses. It can be hypothesized that when individuals consciously recognize an ongoing stressor, distinct response patterns emerge across genders, likely due to the activation of gender-specific coping strategies, such as increased caution or avoidance in one group versus greater risk-taking in the other. In contrast, when stress remains unnoticed, both males and females may exhibit similar risk-taking behaviors, as their responses are guided by more generalized mechanisms rather than gender-specific strategies. Testing this hypothesis could have important implications for understanding the relationship between stress and risk-taking behavior. If risk responses under stress are indeed influenced by the awareness (vs. the lack of awareness) of the stressor, it would suggest that coping strategies are not merely determined by the presence of stress itself but by how individuals perceive and cognitively appraise it. This would add a whole new perspective in interpreting the results found in the literature on gender differences under acute stress by shifting the focus from stress exposure alone to the key role of the perception and cognitive processing of stressors.

**Fig. 1:**
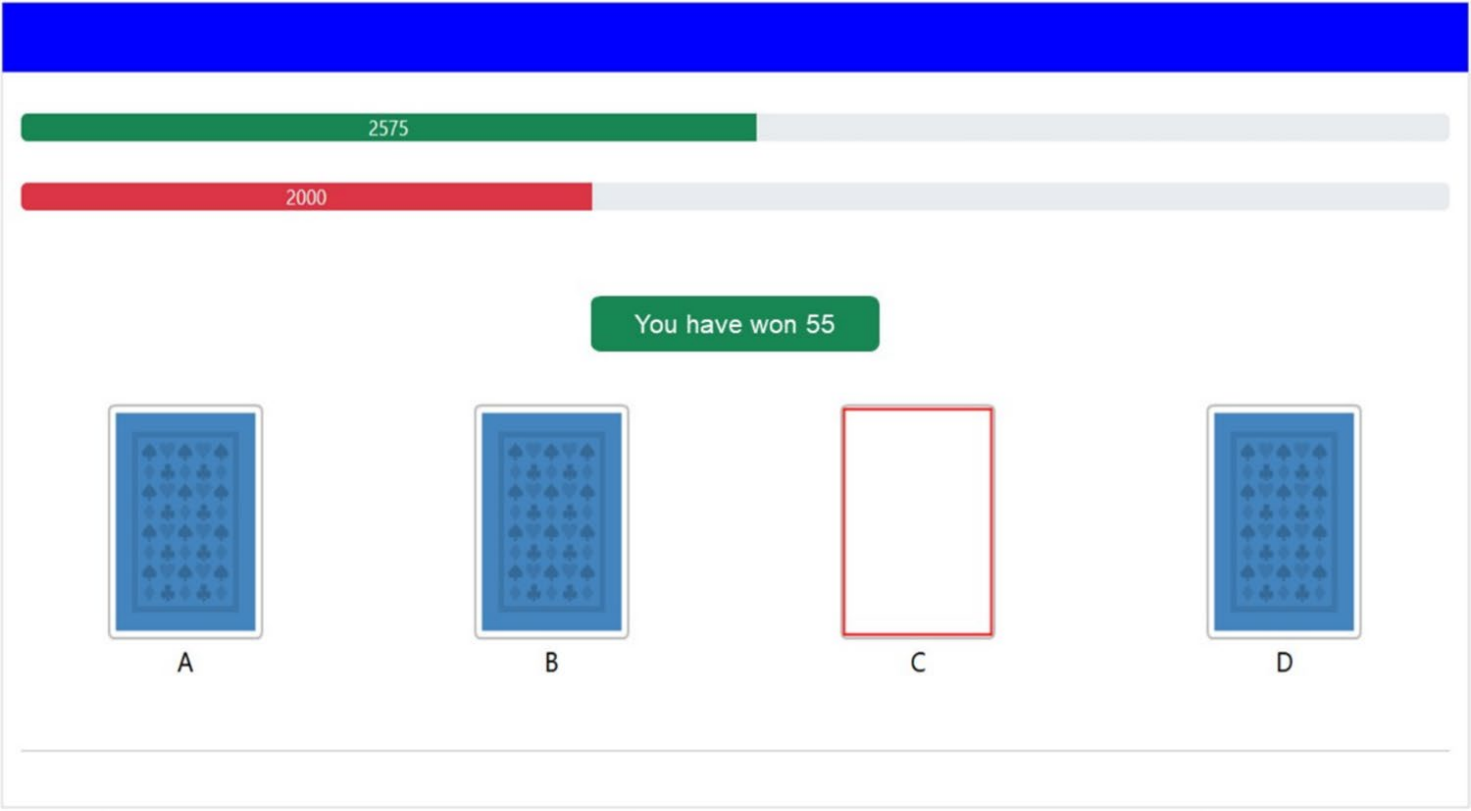
Computerized version of the Iowa Gambling Task employed in the present study. Example of trial in which the participant picked a card from Deck C and received a reward of 55 points. The difference between the two progress bars indicates the participant’s current winning

Thus, the aim of the present study was to examine how the manipulation of stress awareness influences gender differences in decision-making under uncertainty. Specifically, we investigated whether being aware (or unaware) of experiencing stress alters the way males and females make decisions in ambiguous contexts, where the outcome probabilities are not known. The present investigation aimed at understanding how stress, and the awareness associated with it, may differentially impact decision-making behavior across genders. To assess decision-making behavior under uncertainty, we used the Iowa Gambling Task (IGT; Bechara et al., 1994), a widely employed experimental paradigm designed to evaluate decision-making abilities within a controlled laboratory environment.^1^ The task, which simulates a card game with real monetary rewards and penalties, requires complex decision-making based on probabilistic learning, as participants navigate between advantageous and disadvantageous decks to maximize long-term gains (see Fig. 1). Indeed, optimal task performance demands that participants prioritize long-term gain over short-term rewards, thus avoiding significant losses (see more details in the *Method* section). Previous studies have shown that male and female participants’ performances in this task were differentially affected by acute, aware stress, with female participants performing better under stress than male participants (Preston et al., 2007; van den Bos et al., 2009).

As in Pighin et al. (2020), in the present study participants were exposed to mild hypoxia (i.e., reduced oxygen availability), a systemic stressor that induces a physiological stress response, including increased cortisol levels and alterations in autonomic nervous system activity (Bärtsch & Gibbs, 2007). While mild hypoxia is a common environmental stressor at high altitudes (e.g., during mountain climbing and air travel), it also occurs in various work environments in which oxygen levels are intentionally reduced for specific purposes (e.g., reducing oxidative degradation, preventing fires, or enhancing physical training and rehabilitation in athletes). Additionally, mild oxygen deficiency is a common hazard in industries where workers operate in confined spaces with limited or no natural ventilation (Burtscher et al., 2012). One of the most intriguing, yet problematic, aspects of mild hypoxia is that it exerts its effects without conscious recognition (Herman et al., 2003): Individuals experiencing mild hypoxia are often unaware that they are in a situation that compromises their body’s normal functioning (see also Pighin et al., 2012, 2014). This virtually unique characteristic makes mild hypoxia a valuable experimental setting for investigating the role of stress awareness in decision-making. If gender differences in decision-making under stress depend on the awareness that a stressor is present, such differences should only emerge when participants are informed about being subjected to mild hypoxia. Conversely, if these differences persist regardless of awareness, it would suggest a more fundamental, physiological basis for gender-specific stress responses.

## Methods

### Research design

The present study employed a mixed experimental design combining within-subjects and between-subjects factors. Oxygen availability was manipulated within-subjects: each participant was exposed to two distinct conditions, mild hypoxia (FiO_2_ = 14.1%) and normoxia (FiO_2_ = 20.9%), in separate sessions. In contrast, stress awareness was manipulated between-subjects: half of the participants were informed about the oxygen condition they were experiencing, while the other half received no such information. Participants’ gender was treated as a control variable, with a balanced representation of males and females in each experimental group. All participants completed the IGT under both mild hypoxic and normoxic conditions.

### Participants

To determine the sample size needed for the present study, an a priori power analysis was conducted employing a simulation approach (Kumle et al., 2021) implemented in *R* (R Core Team, 2022). Given that, in addition to main effects, the effect of interest was the interaction between oxygen manipulation, awareness of the manipulation, and gender, and that detecting interactions might require larger samples compared to main effects (e.g., da Silva Frost & Ledgerwood, 2020), the sample size needed to detect a small effect (i.e., log odds ratio = 0.5, corresponding to Cohen’s *d* = 0.28; Sánchez-Meca et al., 2003) with at least 80% power was computed. The analysis suggested that a sample of 60 participants performing 200 trials (i.e., 100 in the normoxic session and 100 in the mild hypoxic session) would provide 86% power to detect the interaction effect. Accordingly, a sample of 71 participants was recruited to accommodate potential data exclusions. Seven individuals did not complete the protocol (i.e., they were unable to attend one of the three sessions for upcoming personal reasons), and were consequently excluded from the study.

The final sample comprised 64 participants (53% female, 47% male; *M*_age_ = 22.9 years; *SD* = 3.4 years). The study received approval from the Research Ethics Committee of the University of Trento (Trento, Italy, Prot. No. 2022–050). Individuals with a history of heart conditions (e.g., cardiovascular disease, angina, heart attack) were excluded due to the potential adverse effects of mild hypoxia on heart function (West, 2017). Participation was contingent on providing written informed consent.

### Procedure

The experiment was conducted in the hypoxic chamber of the Sport, Mountain, and Health Research Center (CeRiSM) of the Universities of Verona and Trento (Italy) in Rovereto (204 m above sea level). This facility enables the creation of a controlled normobaric hypoxic environment by adjusting the fraction of inspired oxygen (FiO₂) through an oxygen dilution system based on the vacuum-pressure swing absorption principle (B-Cat, Tiel, The Netherlands). This system introduces oxygen-depleted air into the chamber while maintaining constant total pressure (i.e., normobaric hypoxia).

To recruit participants, flyers were distributed in public and university areas, inviting volunteers to take part in a study on decision-making. Those who expressed interest contacted the research team via email, and an appointment was scheduled for each. Participants were convened in groups of three at a time, with the same group composition maintained across sessions. Additionally, the groups were gender-homogeneous: each group consisted exclusively of all males or all females, and the participants did not know each other prior to the experiment. During the sessions, participants were seated at tables separated by dividers, designed to isolate them from one another and minimize potential distractions or social interactions. Each participant was always seated in the same spot across all sessions, ensuring consistency in the experimental environment and further reducing possible sources of variability.

Each participant was scheduled for three sessions, spaced 1 week apart, to ensure adequate time between the phases of the experiment. The first session was dedicated to familiarization with the experimental environment and procedures. This phase was introduced to reduce the effect of stress related to novelty, ensuring that any responses observed in subsequent sessions were attributable to the experimental conditions and not to the novel environment. During this session, participants received detailed instructions and had the opportunity to familiarize themselves with the IGT, with the data collected not being used for final analyses. The two subsequent sessions constituted the core of the experiment. In each of these sessions, participants were exposed to one of the two experimental conditions: mild hypoxia (FiO_2_ equal to 14.1%, equivalent to an altitude of 3,000 m/9,842 ft.) or normoxia (FiO_2_ equal to 20.9%, i.e., the control condition). The order of sessions was counterbalanced: half of the participants started with hypoxia and then moved to normoxia the week after, while the other half followed the reverse order. This approach allowed for control over potential effects related to the order of session presentation. All sessions were conducted in the same chamber, with a stable temperature of 21°C and air humidity maintained at 32%.

Half of the participants were informed about the oxygen session they were in, while the other half received no information about it. Information was given at the beginning of the session. Those receiving the information were told they were in a “normal oxygen session” or in a “partially decreased oxygen session” according to the session. Those receiving no information about the presence of mild hypoxia were told that they could find themselves in a mildly hypoxic environment (simulating an altitude of 3,000 m above sea level) in some of the sessions, in all of the sessions, or in none of the sessions.

At the onset of each session, participants were instructed to view a 20-min documentary to allow ample time for the physiological changes associated with mild hypoxia to manifest. Subsequently, participants engaged in the IGT (Bechara et al., 1994; see description below) using a tablet and headphones.

The technical apparatus utilized for measuring heart rate was applied at the commencement of each session, and heart rate was recorded at 1-s intervals using a Polar H10 sensor (Polar, Kempele, Finland). Arterial oxygen saturation (SaO_2_) and mean arterial pressure (MAP) were measured by means of portable devices: Nonin WristOx2 3150 (Nonin Medical Inc. Minnesota, USA) and Omron M3 (Omron, Mannheim, Germany), respectively. SaO_2_ and MAP were monitored at three specific time points during each session: following the conclusion of the video clip (approximately 25 min after entering the testing environment), after completion of the Iowa Gambling Task (around 35 min after entering the testing environment), and prior to exiting the room.

At the end of the IGT, participants completed a subjective feelings questionnaire, partially adapted from the Hospital Anxiety and Depression Scale (HADS), which included seven specific anxiety-related items pertaining to participants’ experience in the session they were in (see Table 1). Moreover, before leaving the room, participants in the unaware condition were prompted to identify which session (normoxic or mild hypoxic) they believed they had been in.

**Table 1:** The subjective feelings questionnaire. For each item, participants could respond on a seven-point scale ranging from 1 “Not at all” to 7 “Extremely”

Number	Item
Q1	I feel tense, restless
Q2	I sense fear, as something negative is going to happen
Q3	Worrying thoughts keep buzzing around in my head
Q4	I can sit here and feel relaxed
Q5	I have a strange feeling, like butterflies in my stomach
Q6	I feel restless, as I should stay in movement
Q7	I feel panic

At the end of the last session, participants were asked two questions designed to assess gender role stereotypes related to risk-taking behavior. The questions were phrased as follows: “In general, how do you perceive [women/men]: as individuals who are willing to take risks or as those who tend to avoid risks?” The order of the two questions was randomized across participants and responses were recorded on a scale from 0 to 10, where 0 indicated “not willing to take risks at all” and 10 indicated “very willing to take risks.”

At the end of the study, participants were thanked, rewarded, and fully debriefed on the objectives of the study. The financial reward was based on participants’ performance in the IGT. To prevent potential portfolio effects and strategic behavior across sessions, they were informed that only one of the three sessions would be randomly selected for payout. This approach ensured that participants remained fully engaged and motivated in each session, as their performance in every session had an equal chance of determining their final reward. Additionally, it minimized any attempt to distribute risk or adjust their decision-making strategies based on cumulative earnings.

### The Iowa Gambling Task

Participants received an initial endowment to start the task and had to maximize their profits over the course of 100 trials by selecting cards from one of four decks (see Fig. 1). Among these decks, two were advantageous, offering smaller immediate rewards but also smaller probabilistic losses, resulting in an overall positive net value. Conversely, the remaining two decks were disadvantageous, providing higher immediate rewards but also higher losses, resulting in an overall negative net value. Although the higher immediate rewards of the disadvantageous decks may be tempting, consistently choosing these decks leads to monetary losses over time, ultimately making them poor choices.

Considering the repeated-measures design of the current study, it was necessary to employ a version of the IGT that could mitigate potential practice effects and performance improvements over time. To this end, we utilized the A-IGT, K-IGT, and Q-IGT versions (administered in this specific order across the three sessions), as tested by Xiao et al. (2013). While all three versions share the same structure in terms of immediate rewards and probabilistic long-term losses, they differ in the proportion of inconsistent reward contingencies, resulting in progressively increasing levels of difficulty. Specifically, the A-IGT is the least complex, the K-IGT introduces a moderate level of difficulty, and the Q-IGT is the most challenging. As demonstrated by Xiao et al. (2013), differences in the spatial arrangement of the decks and in the underlying reward-punishment structure limit the applicability of previously learned strategies and require participants to re-learn the task through trial and error in each session. This approach preserves the ambiguity-based nature of the task and minimizes the likelihood of strategic transfer across sessions.

The task was implemented in O-Tree, an open-source platform for behavioral research, and administered to participants using tablet devices.

### Data analysis

All analyses were performed using *R*. Physiological parameters across the oxygen sessions were compared using a repeated-measures ANOVA, with gender and awareness conditions included as between-subject factors. A McNemar’s test was employed to compare the paired nominal data regarding participants’ recognition of the oxygen manipulation in each session in the unaware condition.

Participants’ performance in the IGT was analyzed using two measures. First, the net score (an aggregate measure computed as the difference between the number of advantageous deck selections and the number of disadvantageous deck selections) was used to describe overall performance. To examine learning trajectories, the 100 card selections were divided into five blocks of 20 trials each, and group mean net scores were plotted across these blocks. This approach allowed us to visualize how performance evolved over time under different experimental conditions.

Second, participants’ choices at each trial were used to evaluate the effects of the experimental manipulations on decision-making. Dichotomous trial-level choices (advantageous vs. disadvantageous decks) were then analyzed using generalized linear mixed models (GLMMs), which accounted for both within-participant and between-subject variability. GLMMs are particularly suited for this type of repeated-measures design, as they increase statistical power, and preserve the trial-by-trial structure of the responses without reducing them to aggregate scores (as is the case for net scores).

The first GLMM (GLMM_1_) was fitted to evaluate whether deck selection (advantageous vs. disadvantageous) was affected by oxygen session (normoxic vs. mild hypoxic; within-subjects), awareness condition (aware vs. unaware; between-subjects), participant gender (male vs. female; between-subjects), and their interactions, with random intercepts for participants. To determine whether the order in which participants encountered the two sessions affected the results, session order (normoxic-mild hypoxic vs. mild hypoxic-normoxic) was included in the model as a fixed effect dichotomous predictor as well. GLMM_1_ was fitted using the full set of participants’ responses (i.e., 100 choices per session).

Next, for exploratory purposes, to distinguish the impact of aware versus unaware stress on learning the contingencies from its impact on risk preferences after the contingencies were learned, the model was refitted twice: (1) GLMM_2_ included only participants’ responses in blocks 1, 2, and 3 (trials 1–60), representing the learning phase; (2) GLMM_3_ included participants’ responses in blocks 4 and 5 (trials 61–100), representing the performance phase. Significant interactions between factors were analyzed by means of post hoc tests, adjusting *p*-values for multiple comparisons with Bonferroni correction.

A composite average measure was computed for the seven items assessing subjective feelings (Cronbach’s α = 0.772), with higher values indicating greater anxiety. This measure served as the dependent variable in a repeated-measures ANOVA to examine whether feelings varied significantly as a function of oxygen session, gender, and awareness condition. Participants’ responses to questions about gender role stereotypes in risk-taking behavior were analyzed using a repeated-measures ANOVA, with gender as a between-subjects factor. A gender stereotype index was calculated for each individual by subtracting the score assigned to women from the score assigned to men. Higher values on this index indicate a stronger perception of men as more risk-prone than women, reflecting a greater endorsement of this gender role stereotype. Finally, for exploratory purposes, Pearson correlation coefficients were computed to assess the relationship between each participant’s gender stereotype index and their behavioral responses in the IGT in the normoxic and mild hypoxic session, separately for the aware and unaware condition.

## Results

### Manipulation checks

Table 2 reports the descriptive statistics (mean and standard deviation) for the three physiological parameters and for the subjective feeling ratings by awareness condition and gender. With reference to the heart rate (HR), the results of the ANOVA revealed a main effect of oxygen session [*F*(1,60) = 14.87, *p* < 0.001, *η*_*p*_^*2*^ = 0.20], and a main effect of gender [*F*(1,60) = 4.29, *p* = 0.043, *η*_*p*_^*2*^ = 0.07]. HR was higher in the mild hypoxic session compared to the normoxic session (M_MH_ = 77.8 ± 9.7 vs. M_N_ = 73.9 ± 11.8 beats per minute (bpm)). Female participants showed higher HR than male participants across conditions (M_f_ = 78.4 ± 9.4 vs. M_m_ = 72.9 ± 10.1 bpm). No effect of awareness condition and no interactions between factors were observed on HR (all *p*-values > 0.05).

**Table 2:** Mean (and SD) of participants’ physiological parameters and subjective feelings responses by condition and gender

Physiological parameters	Aware condition				Unaware condition			
	Males		Females		Males		Females	
	N	MH	N	MH	N	MH	N	MH
Hear rate	69.7 (12.3)	73.9 (10.5)	76.5 (9.7)	79.2 (8.2)	72.8 (11.1)	76.4 (9.2)	76.5 (12.8)	81.3 (9.4)
SaO_2_	96.8 (1.1)	88.5 (3.2)	97.5 (1.0)	90.7 (1.9)	96.1 (1.5)	88.0 (1.4)	97.7 (.87)	90.9 (2.8)
MAP	82.7 (8.1)	83.0 (8.0)	82.9* (8.1)	82.9 (7.4)	84.5 (4.0)	85.1 (4.2)	82.3 (4.0)	82.1 (9.7)
Subjective Feelings	1.74 (0.7)	2.07 (0.8)	1.61 (0.6)	1.62 (0.6)	1.86 (0.8)	1.79 (0.4)	1.51 (0.4)	1.96 (0.8)

^*^ Due to a technical issue, the mean arterial pressure (MAP) of one participant in this condition was not recorded

*N* normoxic session, *MH* mild hypoxia session, *SaO*_*2*_ arterial oxygen saturation

With reference to oxygen saturation (SaO_2_), a main effect of oxygen session [*F*(1,60) = 486.84, *p* < 0.001, *η*_*p*_^*2*^ = 0.89], and a main effect of gender [*F*(1,60) = 26.35, *p* < 0.001, *η*_*p*_^*2*^ = 0.31] were observed. SaO_2_ was lower in the mild hypoxic session compared to the normoxic session (M_MH_ = 89.6 ± 2.7 vs. M_N_ = 97.1 ± 1.21%). Male participants had significantly lower SaO_2_ than female participants across conditions (M_m_ = 92.4 ± 1.4 vs. M_f_ = 94.2 ± 1.4%). No effect of awareness condition and no interactions between factors were observed on SaO_2_ (all *p*-values > 0.05).

No significant difference was observed in participants’ MAP between the two experimental sessions (M_MH_ = 83.12 ± 7.71 vs. M_N_ = 82.97 ± 6.35 mmHg), between male and female participants or between awareness conditions (all *p*-values > 0.05).

Participants in the unaware condition were not able to identify the oxygen session they were in, with their responses not differing from chance level (McNemar test, *p* = 0.607). Among the 30 participants in this condition, ten correctly identified both the normoxic and the mild hypoxic sessions, five failed to identify either, nine correctly identified the normoxic session but misclassified the mild hypoxic session, and six correctly identified the mild hypoxic session but misclassified the normoxic one.

### IGT performance

**Fig. 2:**
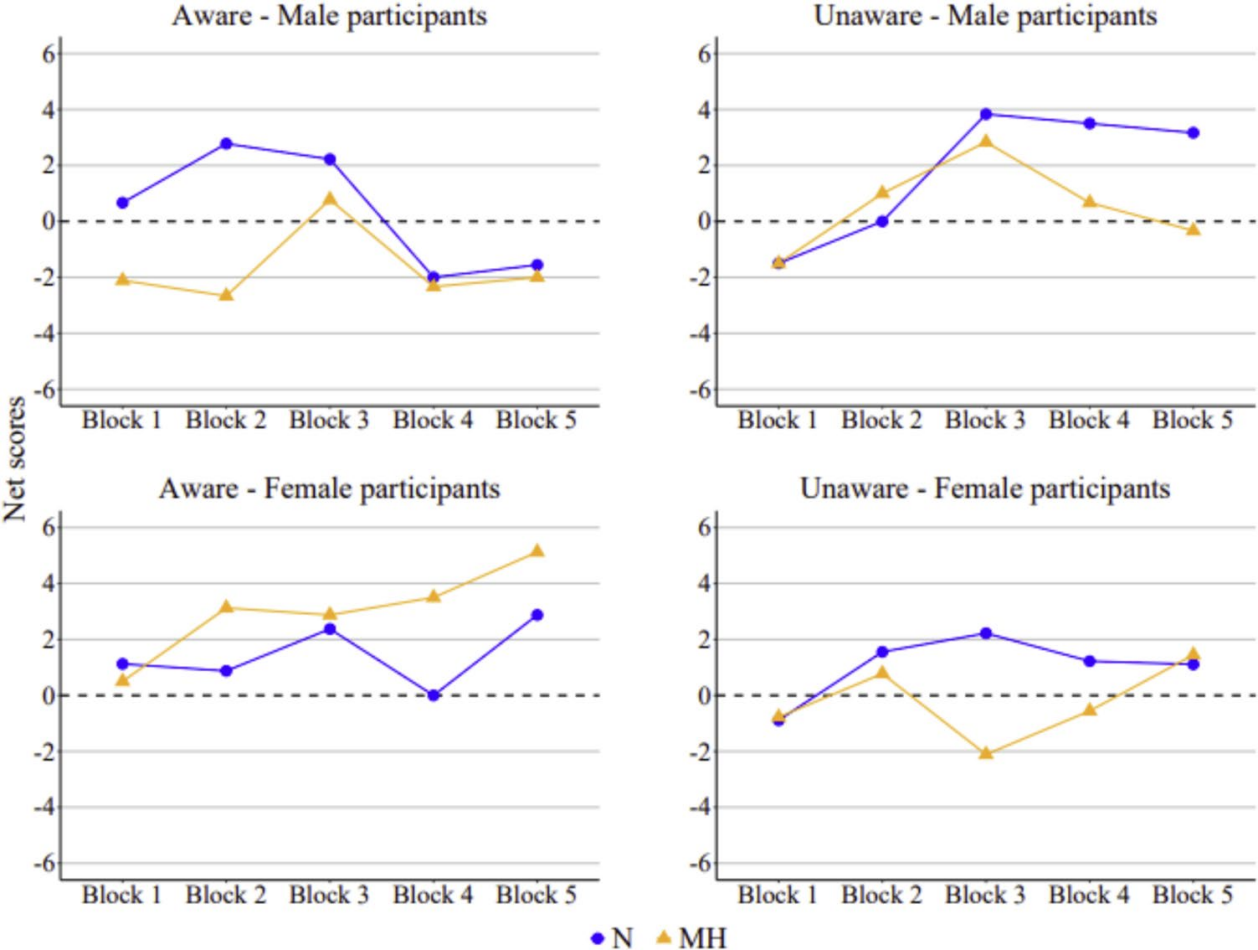
Mean net scores by block in male and female participants across sessions (N = normoxia, MH = mild hypoxia) and awareness conditions

Participants’ performances in terms of total net scores are reported in Fig. 2. A net score above zero implies that participants selected cards advantageously, showing greater sensitivity to large losses and/or a smaller attraction for large rewards; conversely, a net score below zero implies disadvantageous selections, showing a smaller sensitivity to large losses and/or a greater attraction for large rewards (ultimately signifying increased risk-taking).

As can be observed in the four panels of Fig. 2, the learning curve across blocks did not follow a linear increase across virtually all groups,^2^ and, overall, participants tended to show lower net scores in the mild hypoxic session compared to the normoxic session (*M*_MH_ = 1.59, *SD* = 25.1, and *M*_N_ = 5.56, *SD* = 25.0, respectively, aggregating the five blocks). An exception to this trend was seen in the performance of female participants who were aware of the oxygen manipulation (bottom left panel). Indeed, in the mild hypoxic session, these participants not only showed higher net scores, but also gradually switched their preferences toward the advantageous decks (i.e., away from the disadvantageous decks), resulting in roughly increasingly positive scores across the blocks.

**Fig. 3:**
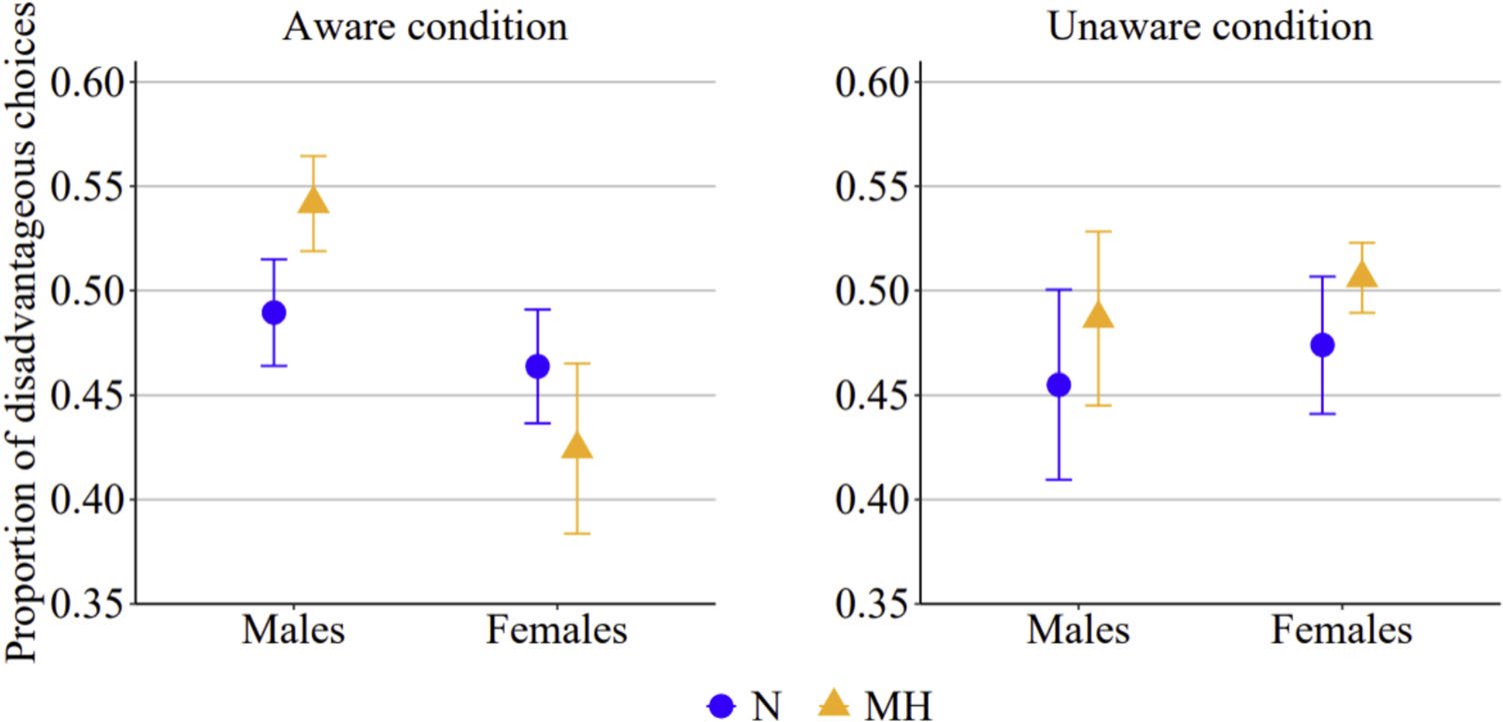
Proportions of disadvantageous choices in the aware and in the unaware condition, in male and female participants when they were in the normoxic (N) or in the mild hypoxic (MH) session, when considering all trials (i.e., 100 per session)

The main statistics of GLMM_1_, GLMM_2_, and GLMM_3_ are reported in Table 3.^3^ For GLMM_1_, the model values of AIC and BIC were 17,400 and 17,474, respectively; the log-likelihood value was −8,690.1, R2 marginal 0.006 and R^2^ conditional 0.048. Session order was not a significant predictor of the probability of choosing the disadvantageous decks (*p* = 0.324); therefore, this variable will not be further discussed. Consistent with the pattern observed with net scores, participants’ choices were influenced by the oxygen manipulation. Overall, the probability of choosing the disadvantageous decks was higher in the mild hypoxic than in the normoxic session (*p* = 0.031). Importantly, however, we found a significant three-way interaction between session, awareness, and gender (*p* = 0.010). As shown in Fig. 3, male and female participants exhibited different behavioral patterns depending on whether they were aware of the oxygen manipulation (left panel) or unaware (right panel). Post hoc comparisons indicated that, when aware of the oxygen manipulation, males were significantly more likely to choose the disadvantageous decks in the mild hypoxic session than in the normoxic session (exp(*B*) = 0.806; 95% CI, 0.653–0.996; *p* = 0.042). Moreover, in the aware condition, male participants in the mild hypoxic session were significantly more likely to choose the disadvantageous decks than females (exp(B) = 1.627; 95% CI, 1.027–2.579; p = 0.027), while in the normoxic session the choice of disadvantageous decks did not differ between males and females (exp(*B*) = 1.112; 95% CI, 0.703–1.762; *p* > 0.05). Female participants tended to decrease the choices of disadvantageous decks under mild hypoxia, but this difference did not reach significance after correcting for multiple comparisons. Conversely, when the oxygen manipulation was unaware, participants, independently from their gender, chose disadvantageous decks slightly more frequently under mild hypoxia (*M* = 0.50, *SE* = 0.02) than under normoxia (*M* = 0.47, *SE* = 0.03), but the contrast failed to reach statistical significance (*p* = 0.081).

**Table 3:** Main statistics of the three generalized linear mixed models (GLMMs) used to analyze behavioral responses on the Iowa Gambling Task (IGT)

Variables		Estimate	SE	Exp(B)	95% Exp(B) CI		z	p
					Lower	Upper		
GLMM_1_ (all blocks)								
(Intercept)		−0.087	0.051	0.916	0.83	1.01	−1.709	0.087
Session	Hypoxia–Normoxia	0.079	0.037	1.082	1.01	1.16	2.159	0.031
Awareness	Unaware–Aware	0.010	0.102	1.010	0.83	1.23	0.095	0.924
Gender	Female–Male	−0.104	0.102	0.901	0.74	1.10	−1.015	0.310
Session Order	Hypoxia/Normoxia–Normoxia/Hyppxia	−0.100	0.101	0.905	0.74	1.10	−0.987	0.324
Session Awareness	Hypoxia–Normoxia Unaware–Aware	0.108	0.073	1.114	0.97	1.27	1.474	0.140
Session Gender	Hypoxia–Normoxia Female–Male	−0.192	0.073	0.826	0.72	0.95	−2.616	0.009
Awareness Gender	Unaware–Aware Female–Male	0.386	0.204	1.471	0.99	2.20	1.888	0.059
Session Awareness Gender	Hypoxia–Normoxia Unaware–Aware Female–Male	0.378	0.146	1.459	1.10	1.94	2.580	0.010
GLMM_2_ (blocks 1–3)								
(Intercept)		−0.79	0.046	0.924	0.85	1.01	−1.729	0.084
Session	Hypoxia–Normoxia	0.107	0.047	1.113	1.02	1.22	2.288	0.022
Awareness	Unaware–Aware	0.069	0.091	1.072	0.90	1.28	0.761	0.447
Gender	Female–Male	−0.039	0.091	0.961	0.80	1.15	−0.432	0.666
Session Order	Hypoxia/Normoxia–Normoxia/Hyppxia	−0.124	0.090	0.883	0.74	1.05	−1.377	0.169
Session Awareness	Hypoxia–Normoxia Unaware–Aware	−0.045	0.094	0.956	0.80	1.15	−0.481	0.630
Session Gender	Hypoxia–Normoxia Female–Male	−0.118	0.094	0.889	0.74	1.07	−1.259	0.208
Awareness Gender	Unaware–Aware Female–Male	0.224	0.182	1.251	0.88	1.79	1.231	0.218
Session Awareness Gender	Hypoxia–Normoxia Unaware–Aware Female–Male	0.575	0.188	1.778	1.23	2.57	3.067	0.002
GLMM_3_ (blocks 4–5)								
(Intercept)		−0.114	0.085	0.892	0.76	1.05	−1.347	0.178
Session	Hypoxia–Normoxia	0.042	0.060	1.043	0.93	1.17	0.701	0.483
Awareness	Unaware–Aware	−0.078	0.170	0.925	0.66	1.29	−0.460	0.646
Gender	Female–Male	−0.202	0.170	0.817	0.59	1.14	−1.187	0.235
Session Order	Hypoxia/Normoxia–Normoxia/Hyppxia	−0.090	0.168	0.914	0.66	1.27	−0.535	0.593
Session Awareness	Hypoxia–Normoxia Unaware–Aware	0.359	0.119	1.432	1.13	1.81	3.011	0.003
Session Gender	Hypoxia–Normoxia Female–Male	−0.326	0.119	0.772	0.57	0.91	−2.733	0.006
Awareness Gender	Unaware–Aware Female–Male	0.682	0.339	1.978	1.02	3.85	2.012	0.044
Session Awareness Gender	Hypoxia–Normoxia Unaware–Aware Female–Male	0.068	0.236	1.070	0.67	1.71	0.284	0.776

**Fig. 4:**
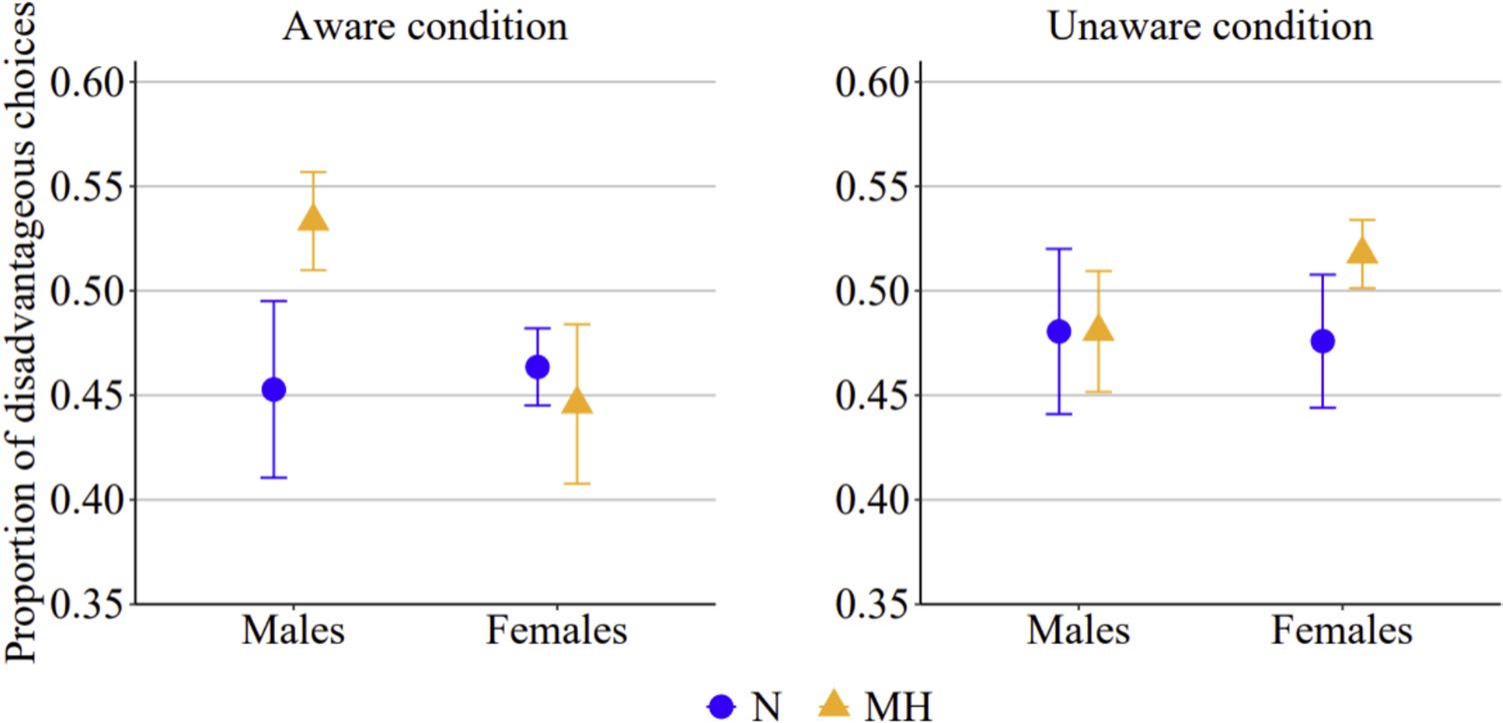
Proportion of disadvantageous choices in the aware and in the unaware condition, in male and female participants when they were in the normoxic (N) or in the mild hypoxic (MH) session, when considering only blocks 1, 2, and 3 (i.e., 60 trials. from 1 to 60)

For GLMM_2_, the model values of AIC and BIC were 10.536 and 10.606, respectively; log-likelihood value was −5258, R^2^ marginal 0.006, and R^2^ conditional 0.033. This second analysis revealed a pattern of results similar to those observed and discussed for GLMM_1_ (see Fig. 4). When considering only the first three blocks (trials 1–60), there was a significant main effect of the oxygen session (*p* = 0.022) and a significant three-way interaction between oxygen session, awareness condition, and gender (*p* = 0.002). Once again, no significant differences were found between male and female participants in the normoxic and mild hypoxic sessions when the oxygen manipulation was unaware. However, when participants were aware of it, males increased their choices of the disadvantageous deck under mild hypoxia compared to normoxia (exp(*B*) = 0.717; 95% CI, 0.703–1.762; *p* = 0.041), while females exhibited the opposite (though not statistically significant) trend.

**Fig. 5:**
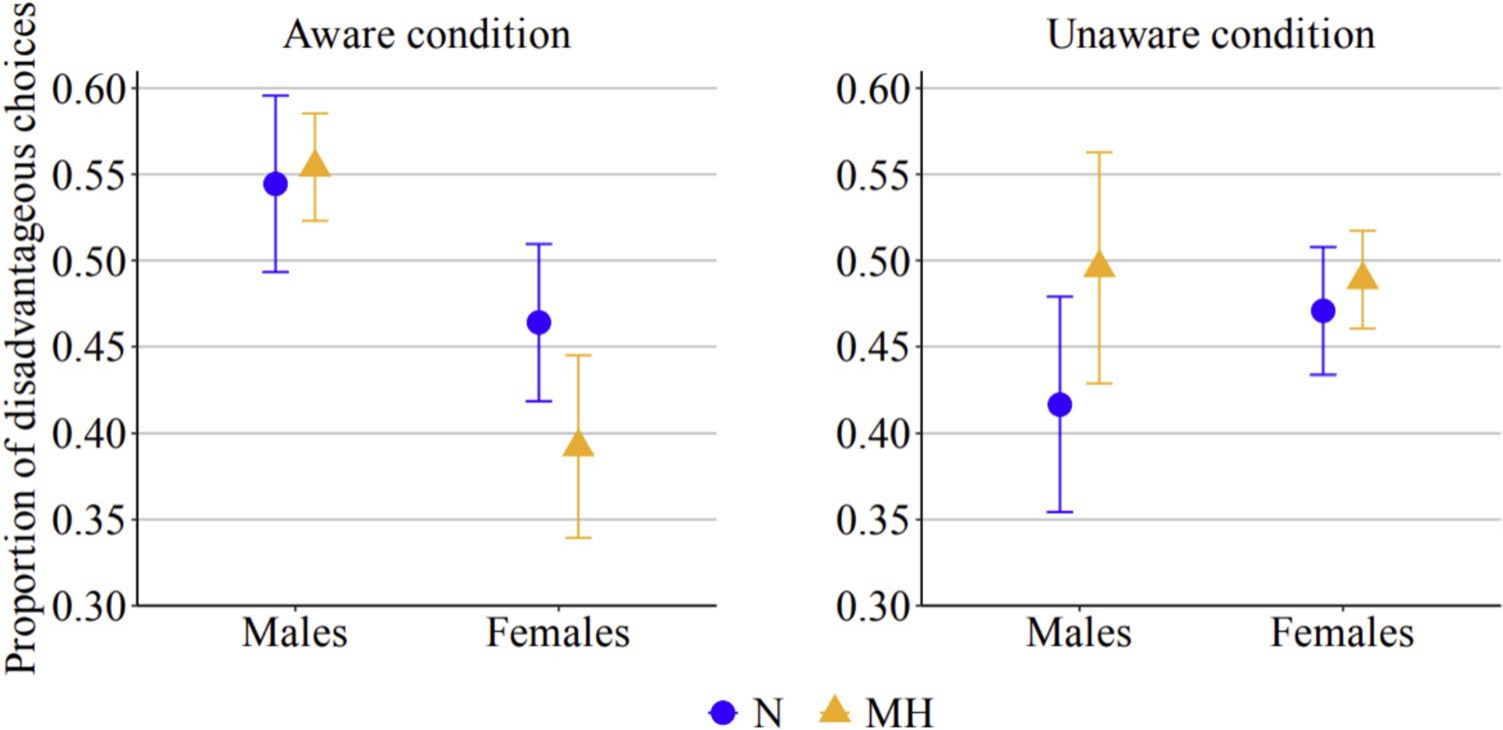
Proportion of disadvantageous choices in the aware and in the unaware condition, in male and female participants when they were in the normoxic (N) or in the mild hypoxic (MH) session, when considering only blocks 4 and 5 (i.e., 40 trials. from 61 to 100)

**Fig. 6:**
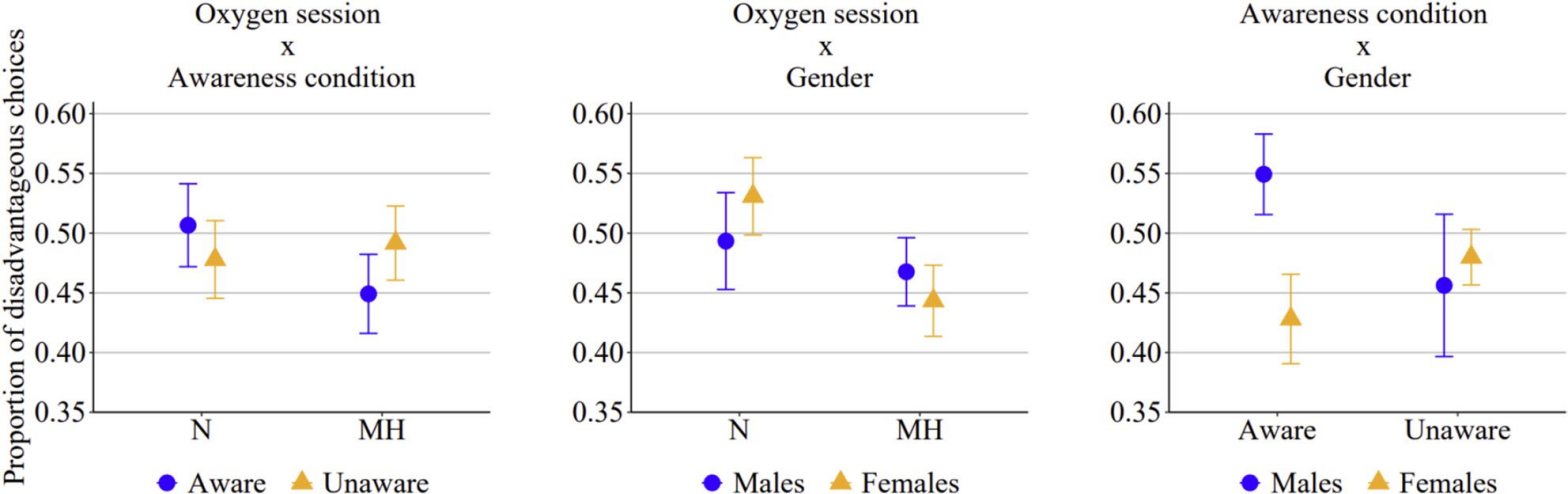
Proportions of disadvantageous choices according the three significant two-ways interactions observed in the second generalized linear mixed model (GLMM_3_) (N = normoxic session; MH = mild hypoxic session)

For GLMM_3_, the model values of AIC and BIC were 6.788 and 6.853, respectively; log-likelihood value was −3384, R^2^ marginal 0.016, and R^2^ conditional 0.120. Unlike the previous two analyses, this third analysis (see Fig. 5) revealed that both the main effect of the session and the significant three-way interaction were no longer present. This may be due to a reduction in statistical power, potentially resulting from a smaller number of analyzed trials, considering only the last two blocks (i.e., 40 trials). Nevertheless, significant interactions emerged between oxygen session and awareness condition (*p* = 0.003), oxygen session and gender (*p* = 0.006), and awareness condition and gender (*p* = 0.044). The interaction between oxygen session and awareness condition revealed that in the normoxic session, participants made slightly more disadvantageous choices in the aware condition compared to the unaware one. In contrast, in the mild hypoxic session, the number of disadvantageous choices was approximately the same in the aware and in the unaware condition (see Fig. 6, left panel). The interaction between oxygen session and gender suggested that, regardless of awareness condition, male participants made more disadvantageous choices than female participants in the mild hypoxic session, whereas no gender differences were observed in the normoxic session (see Fig. 6, central panel). Finally, the interaction between awareness condition and gender indicated that in the unaware condition, the number of disadvantageous choices did not differ between males and females. However, in the aware condition, males tended to make more disadvantageous choices than females (see Fig. 6, right panel).

### Subjective feeling ratings and gender role stereotypes

**Fig. 7:**
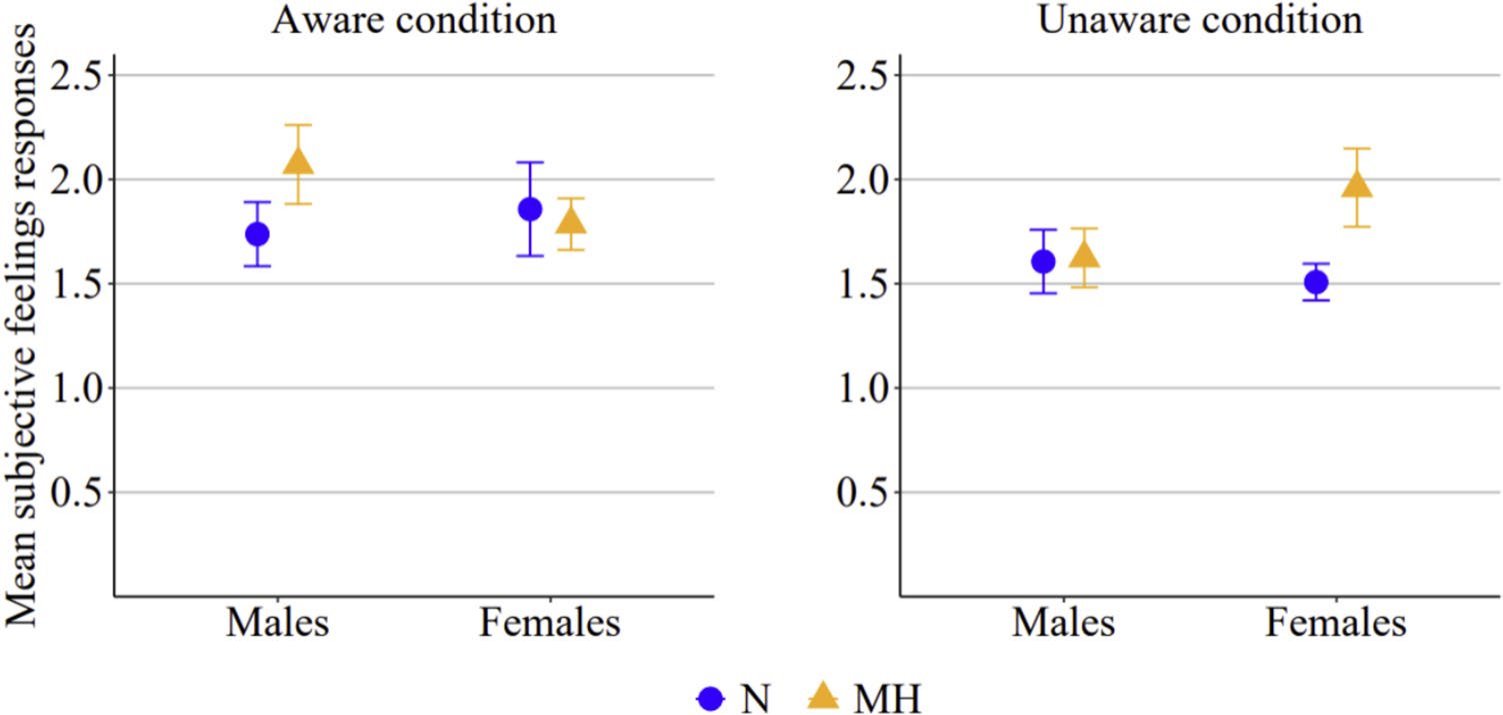
Mean participant responses on the subjective feeling scale across the two oxygen sessions and awareness conditions. Error bars represent the standard error of the mean

The analyses of subjective feelings revealed that, overall, participants reported low levels of anxiety throughout all sessions (*M* = 1.77, *SD* = 0.53). However, a significant interaction effect among oxygen session, awareness condition, and gender was observed [*F*(1,60) = 5.18, *p* = 0.026, *η*_*p*_^*2*^ = 0.08]. As shown in Fig. 7, in the aware condition, male participants reported higher anxiety in the mild hypoxic session compared to the normoxic session, whereas female participants in the aware condition reported similar levels of anxiety across both sessions. In contrast, the pattern was reversed in the unaware condition: male participants reported similar ratings across sessions, whereas female participants reported greater anxiety in the mild hypoxic session than in the normoxic session. However, post hoc comparisons did not remain significant after Bonferroni correction.

Notably, although participants’ subjective feelings showed an interaction between oxygen session, awareness condition, and gender, no corresponding interaction effects were observed in the physiological parameters. This suggests that the reported emotional responses may not reflect genuine differences in physiological arousal, nor are they likely attributable to an uneven distribution, across experimental conditions, of participants characterized by a different propensity to experience negative feelings.

Participants exhibited a significant gender role stereotype in risk-taking behavior [*F*(1,62) = 21.24, *p* < 0.001, *η*_*p*_^*2*^ = 0.26]. Both male and female respondents equally perceived men as more risk-prone than women, as indicated by positive gender role index scores (M_m_ = 0.93 ± 1.36 vs. M_f_ = 0.88 ± 1.74). Interestingly, when participants were aware of the oxygen manipulation, Pearson correlation coefficients revealed that, for male participants, there was a significant positive relationship between the gender role stereotype index and the proportion of disadvantageous choices under both the normoxic (*r*(18) = 0.50, *p* = 0.036) and mild hypoxic (*r*(18) = 0.53, *p* = 0.023) session. In contrast, for female participants, a significant negative correlation emerged between the gender role stereotype index and the proportion of disadvantageous choices under the normoxic session (*r*(16) = −0.52, *p* = 0.037), but no significant relationship was observed under the mild hypoxic session. Therefore, a stronger endorsement of the belief that men are more risk-prone than women seems to be associated with an increase in disadvantageous choices among male participants. Among female participants, however, this belief correlates with a decrease in disadvantageous choices, but only under no-stress conditions. Importantly, when participants were unaware of the oxygen manipulation, no significant correlations were found between these variables (all *p*-values > 0.05).

## Discussion

The present study aimed to investigate whether awareness of acute stress, induced by mild hypoxia, modulates gender differences in decision-making under uncertainty, as measured by the Iowa Gambling Task (IGT).

Physiological data confirmed that the mild oxygen manipulation employed in the present study was effective in altering participants’ HR and SaO_2_ levels. Despite significant alterations in physiological parameters, participants in the unaware condition failed to accurately identify the oxygen levels associated with each session, and this ensures that the oxygen manipulation remained effectively concealed. Behavioral data from the IGT revealed distinct decision-making patterns based on participants’ awareness of the oxygen manipulation. When aware of the environmental stressor, male participants significantly increased their selection of disadvantageous decks under stress (i.e., in the mild hypoxic session) compared to the control condition (i.e., normoxic session), indicating a heightened propensity for risk-taking when stress is consciously perceived; in contrast, female participants who were aware of the ongoing stressor exhibited a modest but consistent shift toward advantageous decks, suggesting a more cautious decision-making style under stress. Thus, when informed about being in a stressful environment, males tended to take more risks, whereas females tended to reduce risk-taking relative to the non-stressful condition. Conversely, in line with the study by Pighin et al. (2020), no significant gender differences emerged when participants were not aware of the stress, with both males and females exhibiting similar, modest increases in disadvantageous choices under a stressful environment compared to the non-stressful condition. These results indicate that decision-making is compromised under stress in a similar way in male and female participants, when they are not aware of being under stress.

Although there were minor variations in effect magnitude, a consistent pattern emerged across both phases of the task. Indeed, in both the learning phase (blocks 1–3) and the performance phase (blocks 4 and 5), the data indicate that awareness of the stressor influenced gender-specific decision-making responses. This consistency suggests that stress awareness impacts how males and females approach risk under stress, rather than exerting an effect limited to either the initial adaptation to the task or the later stages of performance. Overall, these findings highlight the sustained influence of cognitive appraisal on decision-making, reinforcing the idea that the perception of stress, rather than its mere presence, modulates risk-taking behavior differently across genders.

Supporting this interpretation, the analysis of participants’ subjective feelings revealed a noteworthy pattern linked to stress awareness, which reflected distinct gender-related emotional responses. Specifically, when participants were aware of the ongoing stress, males reported elevated levels of anxiety under mild hypoxia relative to normoxia, while females maintained relatively consistent ratings across the two sessions. Conversely, in the absence of stress awareness, females reported higher anxiety under mild hypoxia compared to normoxia, whereas males’ ratings remained stable. Importantly, these interaction patterns were not mirrored in the physiological parameters, which showed no corresponding three-way interaction effects. This dissociation suggests that the observed differences in the experience reported by the participants are more likely attributable to cognitive appraisal processes than to genuine variations in physiological stress responses.

The observed relationship between gender role stereotypes and behavioral responses provides a promising, albeit still incomplete, perspective on how stress awareness shapes gender-specific decision-making. Notably, the findings suggest that gender role stereotypes are associated with risk-taking behavior, particularly in male participants, but only when the stress manipulation was explicitly recognized. In contrast, this association was absent when participants were unaware of the stressor, reinforcing the idea that conscious appraisal of stress may activate gendered behavioral patterns, whereas an unperceived stressor elicits more generalized responses.

Overall, these findings suggest that the mechanisms underlying gender differences in risk-taking under stress extend beyond biological factors alone. Indeed, the activation of gender-specific coping strategies contingent on the cognitive appraisal of stress lends support to the notion that social conditioning and culturally reinforced expectations may play a significant role in shaping specific behavioral responses. For example, women, who are often socialized to prioritize caution and security, may be more inclined to employ conservative, risk-averse strategies when they are aware of stress. This conscious appraisal activates socially reinforced behaviors, leading to a more measured response under ambiguous conditions. In contrast, in the absence of such appraisal, these gender-specific coping mechanisms are less likely to be engaged, resulting in a more uniform pattern of behavior across genders. In this sense, the observed results align with social role theory (Eagly & Wood, 2012), which posits that gender differences in behavior primarily emerge from culturally prescribed roles and expectations.

While the study provides important contributions, some limitations must be acknowledged. First, the a priori power analysis computed for determining the required sample size for the study assumed that each participant would complete 100 trials per oxygen session (i.e., the full task). An investigation aimed at distinguishing the impact of the different factors (i.e., oxygen session. gender. awareness condition) between learning (60 trials) and performance (40 trials) phases would necessitate a different sample size calculation; consequently, our phase-specific analyses should be regarded as exploratory and warrant further investigation with tailored methodologies. Second, the study employs the IGT where risky choices lead to disadvantageous outcomes, yet in other paradigms (such as the BART) riskier choices can yield advantageous outcomes. Thus, our current results may not be generalizable to all forms of decision-making under ambiguous conditions. Third, although the observed interaction effect reached statistical significance, its modest effect size suggests that the magnitude of the observed differences between conditions is relatively small. This implies that while the effect is unlikely to be due to chance, its practical significance may be limited. In other words, the impact of the interaction on decision-making behavior under ambiguous conditions might be subtle in real-world settings. Consequently, these findings should be interpreted with caution, and future research should aim to replicate and extend these results in larger samples or alternative experimental paradigms.

In summary, the present study shows that awareness of an acute stressor (induced by mild hypoxia) modulates decision-making under uncertainty in a gender-dependent manner. These findings not only contribute to a deeper understanding of the mechanisms underlying gender differences in risk-taking behavior but also emphasize how stress appraisal determines cognitive performance, and they may have broader implications for understanding how unaware stress influences decision-making in real-world settings. In everyday life, individuals are often exposed to stressors that may not be consciously perceived but nonetheless alter their cognitive functioning. For example, professionals working in enclosed or poorly ventilated environments (such as pilots in cockpits, or construction workers operating in confined underground spaces) may encounter mild hypoxia or elevated levels of carbon dioxide (CO₂) without being fully aware of their effects. Similarly, first responders or manual laborers working in environments with suboptimal thermal condition without adequate hydration may experience physiological stress due to dehydration, which can impair cognitive performance without the individual recognizing it as stress. Other examples include shift workers exposed to circadian misalignment or individuals experiencing subclinical sleep deprivation. The present findings suggest that increasing awareness of such subtle physiological stressors in occupational and high-stakes environments may be fundamental for promoting safer and more effective decision-making. Future research should continue to explore these dynamics by investigating not only the underlying neurobiological and hormonal mechanisms but also by conducting a more in-depth analysis of the sociocultural factors that may influence stress appraisal and decision-making. Such a comprehensive approach will inform the development of more effective strategies for decision-making in stressful environments but will also refine theoretical frameworks regarding the association between biological predispositions and social conditioning, advancing our overall understanding of adaptive behavior.

## Data Availability

All data generated and analyzed during the current study are available via the Open Science Framework at the following link: https://osf.io/zvqpk/?view_only=f9497779e62c4b19ad31658c1ccefd07

## Footnotes

1 Based on the availability of probabilistic information, uncertainty is frequently divided into risk (situations in which the probabilities of outcomes are known or can be reliably estimated) and ambiguity (situations in which probabilities are undefined or unknown). In this paper, we adopt uncertainty as an inclusive term that reflects the structure of the IGT task, in which participants initially face unknown contingencies and progressively gain experience that allows for probabilistic inference, though probabilities remain implicit throughout.

2 In particular, male participants in the normoxic-aware condition exhibited a descriptively atypical performance pattern during the final phase of the task, characterized by a higher proportion of disadvantageous choices. However, this trend was not statistically significant and may reflect random variation. The underlying causes remain unclear and further investigation is needed to better understand this observation.

3 Reference level for the dependent variable was “disadvantageous choices.” Reference level for the predictors are the second terms in the pairs reported besides the variable names (e.g., for Session, Hypoxia-Normoxia indicates that Normoxia was used as the reference level).

## Code availability

The code used to conduct the statistical analyses is available via the Open Science Framework at the following link: https://osf.io/zvqpk/?view_only=f9497779e62c4b19ad31658c1ccefd07
